# *IL23R* Gene Confers Susceptibility to Ankylosing Spondylitis Concomitant with Uveitis in a Han Chinese Population

**DOI:** 10.1371/journal.pone.0067505

**Published:** 2013-06-28

**Authors:** Hongtao Dong, Qiuming Li, Ying Zhang, Wei Tan, Zhengxuan Jiang

**Affiliations:** 1 Department of Ophthalmology, the First Affiliated Hospital of Zhengzhou University, Zhengzhou, China; 2 Department of Ophthalmology, The First People’s Hospital of Zunyi, The Third Affiliated Hospital of Zunyi Medical University, Zunyi, China; 3 Department of Ophthalmology, the Second Affiliated Hospital of Anhui Medical University, Hefei, China; Oregon Health & Science University, United States of America

## Abstract

**Purpose:**

The interleukin-23 receptor (*IL-23R*) has been shown to be associated with ankylosing spondylitis (AS) in many different populations. This study examined whether *IL-23R* polymorphisms were associated with susceptibility to this disease in a Chinese Han population.

**Methods:**

Three single-nucleotide polymorphisms (SNP), rs7517847, rs11209032, and rs17375018, were genotyped in 291 AS patients and 312 age-, sex-, and ethnically matched healthy controls using a polymerase chain reaction (PCR) restriction fragment length polymorphism (RFLP) assay.

**Results:**

The genotype and allele frequencies of rs17375018, rs7517847, and rs11209032 were not different between the patients with AS and the healthy controls. On the one hand, stratification analysis indicated that the rs17375018 GG genotype and the G allele were increased in AS patients who were *HLA-B27* positive (corrected *p* = 0.024, odds ratio [OR] 2.35, 95% CI 1.30–4.24; *p*_c_ = 0.006, OR 1.98, 95% CI 1.28–3.07, respectively). On the other hand, the analysis according to clinical characteristics showed a significantly increased prevalence of the homozygous rs17375018 GG genotype and the G allele in patients with AS and uveitis compared with the controls (*p*_c_ = 0.024 and *p*_c_ = 0.024, respectively). In addition, haplotype analysis performed with the SHEsis platform revealed no significant difference concerning the haplotypes between AS patients and healthy controls.

**Conclusions:**

In this study, the results suggested that the rs17375018 of *IL23R* was positively associated with *HLA-B27*-positive AS and that the rs17375018 GG of *IL-23R* was associated with AS concomitant with uveitis. We found no evidence for an association between the other two SNPs of IL-23R and AS.

## Introduction

Ankylosing spondylitis (AS) is a chronic inflammatory disease characterized by a diverse spectrum of clinical manifestations, including the alteration of joint architecture, joint fusions, and functional impairment in the sacroiliac and spine joint [1], [2]. The exact pathogenesis and the etiology of AS are not fully understood. Many studies have suggested that genetic factors and certain environmental factors are involved in its development [3], [4], [5]. The idea that genetic factors are strongly implicated in the pathogenesis of this disease is supported by twins having a much higher risk of developing AS [5]. Previous studies revealed that AS was strongly associated with the human leukocyte antigen B-27 allele (*HLA-B27*) in different populations [6], [7]. However, *HLA-B27* only partly accounts for the genetic predisposition to AS. Another study revealed that non-*HLA* genes may be involved in the development of AS [7]. Therefore, studies have been initiated to search for non-*HLA* genes. Studies found that immune-related genes such as endoplasmic reticulum aminopeptidase [8], [9], interleukin-23 receptor (*IL23R*) [10], [11], and interleukin-1 (*IL-1*) [12] were associated with AS in different populations. These results have provided useful information on the genetic predisposition to AS.

As both innate and adaptive immune responses and inflammatory mediators are involved in the pathogenesis of AS [7], molecules involved in the regulation of autoimmunity and inflammation are thought to represent good candidate genes. The interleukin-23 receptor (*IL23R*) gene is located on chromosome 1p31 and highly expressed in dendritic cells [13], [14]. *IL23R* and its ligand, IL-23, are key components of the immune-regulatory pathway. Recently, studies have shown that some single nucleotide polymorphisms (SNPs) of the *IL23R* gene are strongly associated with several autoimmune diseases, such as Crohn’s disease [15], rheumatoid arthritis [16], AS, and Behcet’s disease. Therefore, we wanted to test whether *IL23R* gene polymorphisms are associated with AS in a Chinese Han population.

This case-control study was designed to test the association between specific variants of *IL23R* and the risk for AS. Three SNPs, rs17375018, rs11209032, and rs7517847, were investigated.

## Patients and Healthy Controls

### Study Population

A total of 291 AS patients and 312 healthy controls were recruited from The Third Affiliated Hospital of Zunyi Medical University. Both the patients and the controls were from a Chinese Han population. The control population consisted of unrelated healthy individuals from the same geographical regions as where the AS patients came from, and they were age-, sex-, and ethnically matched with the patients. The patients with AS were diagnosed according to the New York modified criteria [17]. The clinical characteristics of the AS patients were assessed at the time of diagnosis and summarized in Table 1. The study was approved by the local institutional ethics committee of The Third Affiliated Hospital of Zunyi Medical University. All procedures followed the tenets of the Declaration of Helsinki. Written informed consent was obtained from all the subjects. After obtaining the written informed consent, we took 5 ml of peripheral blood from each participant.

**Table 1:** Clinical features of the investigated AS patients and controls.

Clinical features	AS patients		Healthy controls	
	Total (*n* = 291)	%	Total (*n* = 312)	%
Age at onset(years±S.D)	34.6±8.2		37.9±8.4	
Male	165	56.7	169	54.2
Female	126	43.3	143	45.8
HLA-B27	216	69.2	58	16.0
Uveitis	163	56.3	0	
Arthritis	291	100	0	

### SNP Selection and Genotyping

Blood samples were collected in EDTA tubes and kept at −70°C until use. Genomic DNA was extracted from the peripheral blood by the QIAamp DNA Blood Mini Kit (Qiagen, Hilden, Germany). We selected rs17375018 in this study because this SNP was found to be associated with Behcet’s disease in Chinese and Japanese populations [18], [19]. The rs7517847 and rs11209032 SNPs were chosen because they have been shown to be associated with certain immune-related diseases [15], [20]. Amplification of the target DNA was performed by polymerase chain reaction (PCR). The PCR primers and restriction enzymes used in the present study were as described in a recent study [18]. The primers used in this study are presented in Table 2. A 5 µl reaction mixture, which consisted of 2.5 µl Premix Taq (Ex Taq Version; TaKaRa Biotechnology Co. Ltd., Dalian, China), 20 pmoles primers, and 0.2 µg of genomic DNA, was amplified by PCR. The conditions were as follows: initial denaturation at 95°C for 5 min, followed by 38 cycles of denaturation at 94°C for 30 s, annealing at different temperatures (61°C for rs11209032, 55°C for 17375018, and 58°C for 7517847) for 30 s, extension at 72°C for 30 s, and a final extension at 72°C for 5 min. These SNPs were genotyped by PCR restriction fragment length polymorphism (RFLP) analysis. The PCR products of the rs11209032, rs17375018, and rs7517847 polymorphisms were digested with 4 U of XspI (TaKaRa, Dalian, China), BsurI (New England Biolabs, Inc, Ontario, Canada), and Ec0147I (New England Biolabs, Inc, Ontario, Canada) restriction enzymes (Table 2) in a 10 µl reaction volume overnight. The digestion products were visualized on a 3.5% agarose gel and stained with GoldView™ (SBS Genetech, Beijing, China). Direct sequencing was also performed by the Invitrogen Biotechnology Company using randomly selected subjects (20% of all samples) to validate the method used in this study.

**Table 2:** Primers and restriction enzymes used for RFLP analysis of the IL23R gene.

SNP	Primers	Restriction enzyme
rs7517847	5′- CCTTTCACCTATTCCCAAGGCC -3′	ECO147I
	5′- GGGCCTAGGAGACAGCCCATAA -3′	
rs11209032	5′- CTCCCTACATCACCCTCTTTGCACT -3′	XSPI
	5′- TGATAAGGCAATCCGGTGGTTC -3′	
rs17375018	5′- TTTTTCCCATCTTCTTTCTTAA -3′	BSURI
	5′- CGCCCAGCCCTCTTCTCTAATT -3′	

### Statistical Analysis

The Hardy–Weinberg equilibrium (HWE) was tested using the χ^2^ test. The genotype frequencies were estimated by direct counting. The allele and the genotype frequencies were compared between the patients and the controls by the χ^2^ test using SPSS (version 10.0; SPSS Inc., Chicago, IL). Haplotype analysis was performed with the SHEsis platform [21]. The *P* values were corrected (*p*_c_) with the Bonferroni correction by multiplying the *P* value with the number of analyses performed. *p*_c_<0.05 was considered significant.

## Results

The AS patient cohort included 291consecutive subjects (165 male, 126 female), all of whom were from a Chinese Han population. The average age of the patients was 34.6±8.2 years. The healthy control group consisted of 312 subjects (169 male, 143 female), with an average age of 37.9±8.4 years. There was no statistical difference between the AS patients and the controls concerning age and gender. The clinical features of the investigated AS patients and the controls are shown in Table 1.

The results showed that the distribution of the tested *IL23R* SNP genotypes and the alleles did not deviate from the Hardy–Weinberg equilibrium. The genotype and the allele frequencies of the tested *IL23R* SNPs are shown in Table 3. The results revealed that there were no significant differences between the AS patients and the controls concerning the genotype and the allele frequencies of the tested SNPs. As many studies have demonstrated that *HLA-B27* is strongly associated with AS in many different populations, *HLA-B27* may influence the association between the *IL23R* polymorphisms and AS in this study. Therefore, the patients were divided into *HLA-B27*-positive and negative groups. The frequencies of the alleles and the genotypes of the *IL23R* polymorphism in the *HLA-B27*-positive AS patients and the controls are shown in Table 4. The results showed that the frequencies of the rs17375018 GG genotype and the G allele in the AS patients who were *HLA-B27* positive were significantly increased compared to *HLA-B27*-positive controls (*p*_c_ = 0.024, OR 2.35, 95% CI 1.30–4.24; *p*_c_ = 0.006, OR 1.98, 95% CI 1.28–3.07, respectively). Stratification analysis did not show any association of the examined *IL23R* SNPs with the *HLA-B27*-negative patients (data not shown). Haplotype analysis was performed with the SHEsis platform, and no significant difference concerning the haplotypes between AS patients and healthy controls. (data not shown).

**Table 3:** Frequencies of alleles and genotypes of *IL23R* polymorphisms in AS patients and controls.

SNP	Genotype	AS	Controls	χ^2^	*P*	*p*c	OR
	Allele	(*N* = 291)	(*N* = 312)		value		(95% CI)
rs17375018	AA	18(6.2%)	26(8.3%)	1.027	0.311	NS	0.73(0.39–1.35)
	AG	108(37.1%)	136(43.6%)	2.621	0.105	NS	0.76(0.55–1.06)
	GG	165(56.7%)	150(48.1%)	4. 488	0.034	NS	1.41(1.03–1.95)
	A	144(24.7%)	188(30.1%)	4.379	0.036	NS	0.76(0.59–0.98)
	G	438(75.3%)	436(69.9%)	4.379	0.036	NS	1.31(1.02–1.69)
rs7517847	TT	104(35.7%)	98(31.4%)	1.266	0.260	NS	1.21(0.87–1.70)
	GT	146(50.2%)	153(49.0%)	0.077	0.781	NS	1.05(0.76–1.44)
	GG	41(14.1%)	61(19.6%)	3.196	0.074	NS	0.68(0.44–1.04)
	G	228(39.2%)	275(44.1%)	2.968	0.085	NS	0.82(0.65–1.03)
	T	354(60.8%)	349(55.9%)	2.968	0.085	NS	1.22 (0.97–1.54)
rs11209032	GG	53(18.2%)	59(18.9%)	0.048	0.826	NS	0.96(0.63–1.44)
	AG	150(51.5%)	167(53.5%)	0.237	0.627	NS	0.92(0.67–1.27)
	AA	88(30.3%)	86(27.6%)	0.525	0.469	NS	1.14(0.80–1.62)
	A	326 (56.0%)	339(54.3%)	0.346	0.556	NS	1.07(0.85–1.34)
	G	256(44.0%)	285(45.7%)	0.346	0.556	NS	0.93(0.74–1.17)

OR = odds ratio; 95% CI = 95% confidence interval; *p*c = Bonferroni corrected *P*; NS = not significant.

**Table 4:** Frequencies of alleles and genotypes of *IL23R* polymorphism in *HLA-B27-*positive AS patients and controls.

SNP	Genotype	*HLA-B27+*	*HLA-B27+*	χ^2^	*P*	*p*_c_	OR	
	Allele	Patients	controls		value		(95%CI)	
rs17375018	AA	14(6.5%)	8(13.8%)	3.310	0.069	NS		0.43(0.17–1.09)
	AG	71(32.9%)	27(46.6%)	3.725	0.054	NS		0.56(0.31–1.01)
	GG	131(60.6%)	23(39.7%)	8.186	0.004	0.024		2.35(1.30–4.24)
	A	99(22.9%)	43(37.1%)	9.540	0.002	0.006		0.51(0.33–0.78)
	G	333(77.1%)	73(62.9%)	9.540	0.002	0.006		1.98(1.28–3.07)
rs7517847	TT	81(37.5%)	22(37.9%)	0.004	0.952	NS		0.98(0.54–1.79)
	GT	105(48.6%)	30(51.7%)	0.177	0.674	NS		0.88(0.49–1.58)
	GG	30(13.9%)	6(10.3%)	0.503	0.478	NS		1.40(0.55–3.54)
	G	165(38.2%)	42(36.2%)	0.154	0.695	NS		1.09(0.71–1.67)
	T	267(61.8%)	74(63.8%)	0.154	0.695	NS		0.92 (0.60–1.41)
rs11209032	GG	34(15.7%)	11(18.9%)	0.346	0.556	NS		0.80(0.38–1.69)
	AG	124(57.4%)	32(55.2%)	0.093	0.760	NS		1.10(0.61–1.96)
	AA	58(26.9%)	15(25.9%)	0.023	0.880	NS		1.05(0.54–2.04)
	A	240(55.6%)	62(53.4%)	0.164	0.685	NS		1.09(0.72–1.64)
	G	192(44.4%)	54(46.6%)	0.164	0.685	NS		0.92(0.61–1.39)

OR = odds ratio; 95% CI = 95% confidence interval;

*p*_c_ = Bonferroni corrected *P*; NS = not significant.

We further investigated whether the *IL23R* SNPs were associated with certain clinical features of AS. The analysis showed that the frequencies of the rs17375018 GG genotype and the G allele were significantly higher in AS patients with uveitis compared to the controls (*p*_c_ = 0.024 and *p*_c_ = 0.024, respectively). The results are shown in Table 5. The results did not show any association between the other two tested *IL23R* SNPs and uveitis.

**Table 5:** Frequencies of alleles and genotypes of *IL23R* polymorphism in AS patients with uveitis, without uveitis, and controls.

SNP	Genotype	AS patients		Controls	*p*_c_
	allele	withuveitis	withoutuveitis		
rs17375018	AA	10(6.1%)	8(6.3%)	26(8.3%)	
	AG	52(31.9%)	56(43.7%)	136(43.6%)	
	GG	101(61.0%)	64(50.0%)	150(48.1%)	0.024
	A	72(22.1%)	72(28.1%)	188(30.1%)	
	G	254(77.9%)	184(71.9%)	436(69.9%)	0.024
rs7517847	TT	62(38.0%)	42(32.8%)	98(31.4%)	
	GT	77(47.2%)	69(53.9%)	153(49.0%)	
	GG	24(14.7%)	17(13.3%)	61(19.6%)	
	G	125(39.2%)	103(40.2%)	275(44.1%)	
	T	201(60.8%)	153(59.8%)	349(55.9%)	
rs11209032	GG	32(19.6%)	21(16.4%)	59(18.9%)	
	AG	81(49.7%)	69(53.9%)	167(53.5%)	
	AA	50(30.7%)	38(29.7%)	86(27.6%)	
	A	181 (55.5%)	145(56.6%)	339(54.3%)	
	G	145(44.5%)	111(43.4%)	285(45.7%)	

*p*_c_, AS patients with uveitis vs. healthy controls; *p*_c_ = Bonferroni corrected *P.*

## Discussion

Recently, many candidate gene-association studies have been carried out to identify non-*HLA* genes involved in susceptibility to AS. This study investigated whether polymorphisms of *IL23R* contributed to AS in a Chinese Han population. Although there were no significant differences between the AS patients and the controls concerning the genotype and allele frequencies of the tested SNPs, the results showed that rs17375018 in *IL23R* was associated with *HLA-B27*-positive AS. We further investigated whether the *IL23R* SNPs were associated with certain clinical characteristics of AS. The results revealed that rs17375018 was associated with AS concomitant with uveitis.

AS is one of a number of common inflammatory diseases, which result in severe occupational disability as the disease progresses [1]. The development of AS is associated with complex interactions between environmental factors and immune responses [3], [6]. It is clear that genetic factors influence the immune responses and the progression of AS. IL23 is one of the master regulators of immunity. Studies have shown that IL23 promotes inflammatory responses by inducing the production of IL17, IL6, IL8, and tumor necrosis factor-α and that it regulates the amplification and the stability of Th17 lymphocytes [14], [22], which are associated with strong pro-inflammatory responses and severe autoimmunity. Therefore, the IL23 pathway may be involved in the pathogenesis of AS. We selected the *IL23R* gene as a candidate gene mainly based on the following facts: First, *IL23R* is an important component of the IL23 pathway, and the interaction of *IL23R* with its ligand, IL23, can promote the production of IL17, which is known to be involved in many chronic inflammatory diseases [14], [23]. Second, the association between *IL23R* and inflammatory diseases has been extensively studied in recent years [9], [10], [15]. The results of these studies in different populations are controversial and do not specify clearly whether the *IL23R* polymorphism is a risk factor or a protective factor for AS [8], [9], [10], [24]. Third, there is little information on the relationship between the *IL23R* polymorphic variant and the risk of AS in this population. These data prompted us to investigate the association of IL23R polymorphisms and AS in a Chinese Han population.

There are many SNPs in the IL23R gene, and a few are involved in the development of the disease. The rs17375018 SNP was chosen based on a previous study, which showed that this SNP was associated with Behcet’s disease, another common uveitis entity observed in China [18]. The rs7517847 and rs11209032 SNPs were selected as the candidate SNPs mainly because their association with AS, Crohn’s disease, and other autoimmune diseases in different populations has been studied previously [10], [18], [20]. In this study, the results showed that the GG genotype and the G allele of rs17375018 were associated with AS concomitant with uveitis. This result is consistent with that reported in Behcet’s disease in a Chinese Han population and a Japanese population [18], [19]. However, the rs17375018 of IL23R was not associated with Vogt-Koyanagi-Harada syndrome (VKH) in a Chinese population [25]. This study failed to find any association between rs7517847, rs11209032, and AS. Similarly, another study found no association between these SNPs and Crohn’s disease in a Japanese population [26] and no association with VKH and Fuchs’ syndrome in a Chinese population [25], [27]. In contrast, the rs7517847 and rs11209032 SNPs have been reported to be associated with AS in a Spanish population and with Crohn’s disease in a Caucasian population [15], [20]. In common with our findings, a previous study showed that the *IL23R* gene was not associated with AS in a Chinese Han population [8]. Interestingly, when the patients were divided into two groups according to whether they were *HLA-B27* positive or negative, the rs17375018 of *IL23R* was associated with *HLA-B27*-positive AS. This result suggests that *IL23R* may play an important role in the pathogenesis of AS through *HLA-B27*. Further analysis of the clinical features and the *IL23R* polymorphisms suggested that rs17375018 was strongly associated with AS concomitant with uveitis which is an autoimmune disease. It reported that *HLA-B27* is associated with acute anterior uveitis [28], [29], [30]. Taken together, these data suggest that AS concomitant with uveitis and acute anterior uveitis may share a common genetic factor in this population.

Although the current study found an association between *IL23R* polymorphisms and AS concomitant with uveitis, some limitations need to be considered. First, the sample size influenced the power to detect disease susceptibility genes. Second, in addition to the relatively small size, all the subjects came from a Chinese Han population. The results of this study need to be confirmed using large sample sizes and multi-ethnic populations. Extensive studies are needed to clarify the functional role of the *IL23R* gene in the pathogenesis of AS. Additionally, this study only selected three SNPs. Other SNPs of the *IL23R* gene need to be tested in further research.

In summary, our study showed that the rs17375018 of *IL23R* was positively associated with *HLA-B27*-positive AS and that the rs17375018 GG of *IL23R* was associated with AS concomitant with uveitis. We did not find any association between the other two SNPs and AS in this Chinese Han population.
